# Switching and Swapping of Quantum Information: Entropy and Entanglement Level

**DOI:** 10.3390/e23060717

**Published:** 2021-06-04

**Authors:** Marek Sawerwain, Joanna Wiśniewska, Roman Gielerak

**Affiliations:** 1 Institute of Control & Computation Engineering, University of Zielona Góra, Licealna 9, 65-417 Zielona Góra, Poland; R.Gielerak@issi.uz.zgora.pl; 2 Institute of Information Systems, Faculty of Cybernetics, Military University of Technology, Kaliskiego 2, 00-908 Warsaw, Poland; jwisniewska@wat.edu.pl

**Keywords:** local quantum information swapping, quantum qubit switch, entropy, negativity, level of entanglement

## Abstract

Information switching and swapping seem to be fundamental elements of quantum communication protocols. Another crucial issue is the presence of entanglement and its level in inspected quantum systems. In this article, a formal definition of the operation of the swapping local quantum information and its existence proof, together with some elementary properties analysed through the prism of the concept of the entropy, are presented. As an example of the local information swapping usage, we demonstrate a certain realisation of the quantum switch. Entanglement levels, during the work of the switch, are calculated with the Negativity measure and a separability criterion based on the von Neumann entropy, spectral decomposition and Schmidt decomposition. Results of numerical experiments, during which the entanglement levels are estimated for systems under consideration with and without distortions, are presented. The noise is generated by the Dzyaloshinskii-Moriya interaction and the intrinsic decoherence is modelled by the Milburn equation. This work contains a switch realisation in a circuit form—built out of elementary quantum gates, and a scheme of the circuit which estimates levels of entanglement during the switch’s operating.

## 1. Introduction

The constant development of quantum computing manifests itself in a great number of works concerning the physical implementations of quantum computers and networks, but also a wide range of algorithms (e.g., in quantum machine learning) is still evolving. One of the most important fields in quantum computing is analysing and detecting quantum entanglement [1,2,3], which is a characteristic feature of quantum systems. The entanglement [4,5,6], called by Einstein “spooky action at a distance”, plays a significant role in quantum communication protocols. In this article, we present an analysis of entropy and entanglement level during the operation of switching and swapping of two quantum states. We utilise simulation techniques to trace the entanglement in a circuit of known quantum gates, which performs a controlled swapping operation. It should be mentioned that, the results presented in this article on entanglement changes, produced by the switch gate, provide another example of different entanglement levels generated by quantum gates. This phenomenon was also studied in the context of controlled negation gates in [7].

The simplest SWAP is a two-qubit operation, which exchanges the states of two input qubits. We can also encounter the variations of this quantum gate such as controlled SWAP (Fredkin gate) and the square root of SWAP—these gates were proposed in the twentieth century and the basic information concerning them may be found in textbooks raising the subject of quantum computing, for example, [8]. However, the interest in the SWAP gate is still vivid.

The entangling features of the square root of SWAP (and its generalisation: the m-th root of SWAP) are broadly discussed in [9], where the authors emphasise the non-local character of entanglement and estimate the so-called entangling power of unitary operators, which was introduced in [10]. A relationship between SWAP gates and entanglement was also presented in [11], where the circuits built out of controlled Z gates and SWAP gates are optimised. Moreover, the level of entanglement in these circuits is evaluated to check the influence of the mentioned gates and SLOCC (Stochastic Local Operations and Classical Communication) operations on the entanglement.

We can observe the development in the field of SWAP gates implementation. In [12], the optimisation of circuits realising SWAP operation for qudits (generalised units of quantum information) is presented. Physical implementations of the controlled SWAP are shown in [13,14], where, respectively, photonic and superconducting qubit-qutrit circuits are utilised. In 2020, two articles discussed the modern realisations of the SWAP gate. The first one focused on the iSWAP gate built with the use of superconducting circuits [15]. Whereas, in [16], quantum interference patterns were applied to realise the SWAP gate and other controlled two-qubit operations.

Moreover, the Fredkin gate is a component of a circuit called the SWAP-test [17]. This solution allows estimation of the similarity of two quantum states. In the SWAP-test circuit, the phenomenon of entanglement also occurs. The comparison of quantum states is realised as a probability distribution of measuring state |0〉 in one of the circuit’s outputs.

Information swapping is naturally connected with the subject matter of quantum repeaters [18], which were invented to avoid a loss of information through the transmission channel. An entanglement is fragile and its long-distance transfer, which is crucial in communication protocols [19], is problematic to realise. An idea of entanglement swapping has been known since 1993 [20,21] and it enabled the invention of quantum repeaters [22], which swap the entanglement between consequent pairs of qubits in a quantum network to maintain a proper level of the entanglement during the whole process of information transmission.

This paper is organised as follows: in Section 2, the basic information relating to the used mathematical concepts is presented. We describe the SWAP operation for qudits and we define it in terms of a partial trace operation in Section 3. The direct application of the SWAP operation is shown in Section 4—it is presented by the definition of a quantum switch, utilising the SWAP operation. Changes of the entanglement level and von Neumann entropy value during the switch operation are demonstrated for two cases—with and without distortions. The conclusions are captured in Section 5. Acknowledgements and references end the article.

## 2. Preliminaries

In this part of the text, we define the necessary notions and definitions used in our paper. For the convenience of the reader, all used symbols are collected in Table 1.

Let H be a separable complex Hilbert space and let $\left(|\psi_{\alpha }\rangle ,\alpha =1,\ldots \right)$ be a complete orthonormal system (termed as cons) of vectors in H. For any such cons, we define the following operators:

(1) $$|\psi_{\alpha }\rangle \langle \psi_{\beta }|=|\alpha \rangle \langle \beta |=E\left(\alpha ,\beta \right),$$

and in particular

(2) E(α)=E(α,α).

It is easy to observe:

(i) completeness relation:
  (3) $$\sum_{\alpha =1\ldots }E\left(\alpha \right)=I_{H},$$
  where $I_{H}$ means the unity operator in H, and in the case of dim(H)=∞, the sum is strongly convergent.
(ii) orthogonality:
  (4) $$E\left(\alpha \right)E\left(\beta \right)=\delta_{\alpha \beta }E\left(\alpha \right),$$
  where $\delta_{\alpha \beta }$, with lower indices, stands for the corresponding Kronecker symbol,
(iii) orthogonality of operators pairs product:
  (5) $$E\left(\alpha ,\beta \right)E\left(\gamma ,\delta \right)=\delta_{\beta \gamma }E\left(\alpha ,\delta \right),$$
(iv) completeness relation in operator algebra:
  (6) $$\sum_{\alpha ,\beta }E\left(\alpha ,\beta \right)=I_{L_{2}\left(H\right)},$$
  where $L_{2}\left(H\right)$ stands for the Hilbert-Schmidt operators acting on H.

Let $H^{A}$ and $H^{A}$ be separable Hilbert space and let $\left(|\psi_{\alpha }\rangle ,\alpha =1,\ldots \right)$ corr. $\left(|\theta_{\beta }\rangle ,\beta =1,\ldots \right)$ be some cons(s) in $H^{A}$, resp. in $H^{B}$. Then we define:

(7) $$E^{A}\left(\alpha ,\beta \right)=|\psi_{\alpha }\rangle \langle \psi_{\beta }|,\;\;\;E^{B}\left(\gamma ,\delta \right)=|\theta_{\gamma }\rangle \langle \theta_{\delta }|,$$

and similarly for $E^{A}\left(\alpha \right)$ and $E^{B}\left(\gamma \right)$.

From Equation (3) it follows:

(8) $$\sum_{\alpha ,\beta }E^{A}\left(\alpha \right)⊗E^{B}\left(\gamma \right)=I_{H^{A}⊗H^{B}}.$$

In particular, taking some $|\psi \rangle \in H^{A}⊗H^{B}$, we can write:

(9) $$|\psi \rangle =\sum_{\alpha ,\gamma }\psi_{\alpha ,\gamma }|\alpha \gamma \rangle .$$

The swapping operator Sw is defined as:

(10) $$\mathrm{Sw}=\sum_{\beta ,\delta }|\delta \beta \rangle \langle \beta \delta |:H^{A}⊗H^{B}⟶H^{B}⊗H^{A},$$

from which

(11) $$\mathrm{Sw}(|\psi \rangle )=\sum_{\alpha ,\gamma }\psi_{\alpha ,\gamma }|\gamma \alpha \rangle .$$

It is obvious that:

(i) (12) $${\mathrm{Sw}}^{2}={\mathrm{Sw}}^{†}\circ \mathrm{Sw}=I_{H^{A}⊗H^{B}},$$
  where ∘ represents operator composition. From this, it stems that the operator norm of Sw is equal to one which means that Sw is an isometric operator (in fact it is a unitary map).
(ii) if |Ψ〉 is separable: |Ψ〉=|ψ〉⊗|θ〉, then
  (13) Sw(|Ψ〉)=|θ〉⊗|ψ〉.

Let $|\Psi \rangle \in H^{A}⊗H^{B}$ with |||Ψ〉||=1.

Then, applying the Schmidt decomposition (see, e.g. [23,24]) for the dim(H)=∞ case:

(14) $$|\Psi \rangle =\sum_{\lambda =1}^{\infty }\tau_{\lambda }|\omega_{\lambda }^{A}\rangle ⊗|\omega_{\lambda }^{B}\rangle ,$$

where the Schmidt coefficients $\tau_{\lambda }\geq 0$ and $\sum_{\lambda =1}^{\infty }\tau_{\lambda }^{2}=1$, and the systems $\left(|\omega_{\lambda }^{A}\rangle \right)$ and $\left(|\omega_{\lambda }^{B}\rangle \right)$ form cons (complete orthonormal system) in $H^{A}$, resp. in $H^{B}$.

From the very definition Equations (10) and (11), it results that Sw is an isometric operation, therefore its operator norm is equal to one. As the Schmidt decomposition, Equation (14) is convergent in the norm, it follows that, for an arbitrary $|\Psi \rangle \in H^{A}⊗H^{B}$, the action of Sw on |Ψ〉 is given by

(15) $$\mathrm{Sw}(|\Psi \rangle )=\sum_{\lambda =1}^{\infty }\tau_{\lambda }|\omega_{\lambda }^{B}\rangle ⊗|\omega_{\lambda }^{A}\rangle \in H^{B}⊗H^{A}.$$

**Proposition** **1.** *The von Neumann entropy invariance under a swap operation.*

(I) *Let $\min(\dim H^{A},\dim H^{B})=d<\infty$. Then for any pure state |Ψ〉〈Ψ|=Q on $H^{A}⊗H^{B}$, the von Neumann entropy S of the corresponding reduced density matrices:*
  (16) $$Q^{A}\left(\Psi \right)={\mathrm{Tr}}_{H^{B}}\left(|\Psi \rangle \langle \Psi |\right),$$
  *and resp.*
  (17) $$Q^{B}\left(\Psi \right)={\mathrm{Tr}}_{H^{A}}\left(|\Psi \rangle \langle \Psi |\right),$$
  *is an invariant under the action of the Sw operator and*
  (18) $$S\left(Q^{A}\right)=S\left(Q^{B}\right)=S\left(Q^{A}\left(\mathrm{Sw}\left(\Psi \right)\right)\right)=S\left(Q^{B}\left(\mathrm{Sw}\left(\Psi \right)\right)\right).$$
  *In particular:*
  (19) $$Q^{A}\left(\mathrm{Sw}(|\Psi \rangle \langle \Psi |)\right)=U\left(Q^{A}(|\Psi \rangle \langle \Psi |)\right)U^{-1},$$
  *and*
  (20) $$Q^{B}\left(\mathrm{Sw}(|\Psi \rangle \langle \Psi |)\right)=U\left(Q^{B}(|\Psi \rangle \langle \Psi |)\right)U^{-1},$$
  *for some unitary map U.*
(II) *If $\min(\dim H^{A},\dim H^{B})=\infty$ then the same facts, as in point (I), are valid for the renormalised von Neumann entropies defined as*
  (21) $$rS\left(Q^{A}\right)=\sum_{\alpha =1}^{\infty }\left(\tau_{\alpha }^{2}+1\right)\log\left(1+\tau_{\alpha }^{2}\right).$$
  *and similarly for $Q^{B}$.*

**Proof.**  If the Schmidt decomposition of the vector $|\Psi \rangle \in H^{A}⊗H^{B}$ is given by Equation (14), then the corresponding density matrices are given by the formulas:

(22) $$Q^{A}\left(\Psi \right)={\mathrm{Tr}}_{H^{B}}\left(|\Psi \rangle \langle \Psi |\right)=\sum_{\alpha =1}^{d}{\left(\tau_{\alpha }\right)}^{2}|\omega_{\alpha }^{A}\rangle \langle \omega_{\alpha }^{A}|,$$

and, therefore,

(23) $$S\left(Q^{A}\left(\Psi \right)\right)=-\sum_{\alpha =1}^{d}\left(\tau_{\alpha }^{2}\right)\log\left(\tau_{\alpha }^{2}\right),$$

and similarly

(24) $$Q^{B}\left(\Psi \right)={\mathrm{Tr}}_{H^{A}}\left(|\Psi \rangle \langle \Psi |\right)=\sum_{\alpha =1}^{d}{\left(\tau_{\alpha }\right)}^{2}|\omega_{\alpha }^{B}\rangle \langle \omega_{\alpha }^{B}|,$$

and therefore

(25) $$S\left(Q^{B}\left(\Psi \right)\right)=S\left(Q^{A}\left(\Psi \right)\right).$$

Applying Equation (14) and definition of Sw operations, we obtain:

(26) $$Q^{A}\left(\mathrm{Sw}(|\Psi \rangle \langle \Psi |)\right)=Q^{B}\left(\Psi \right),$$

and

(27) $$Q^{B}\left(\mathrm{Sw}(|\Psi \rangle \langle \Psi |)\right)=Q^{A}\left(\Psi \right),$$

from which the equality derives:

(28) $$S\left(Q^{A}\left(\Psi \right)\right)=S\left(Q^{A}\left(\mathrm{Sw}(|\Psi \rangle \langle \Psi |)\right)\right),$$

and similarly

(29) $$Q^{B}\left(\mathrm{Sw}(|\Psi \rangle \langle \Psi |)\right)=Q^{A}\left(\Psi \right),$$

and therefore

(30) $$S(Q^{B}(|\Psi \rangle \langle \Psi |)=S\left(Q^{A}(|\Psi \rangle \right))).$$

Thus, we have presented the validity of (I).

The corresponding Schmidt decomposition theorem in the infinite dimension case is well known, see for example [23]. The corresponding reduced density operators and $Q^{A}$ and $Q^{B}$ do exist and belong to the trace class for any pure state, and their spectrums are identical if we restrict ourselves to the non-zero Schmidt numbers. However, in many cases (see the Remark 1 below) the use of the standard notion of the von Neumann entropy leads to the infinite values. It is due to the fact that the trace of the unit operator is infinite. It is why the concept of the renormalisation of the standard definition of the von Neumann entropy has been introduced by one of us [25], in order to deal with such a situation. For a given reduced density matrix, the corresponding, renormalised entropy is given by the formula:

(31) $$rS\left(Q^{A}\right)=\mathrm{Tr}\left(I_{H_{A}}+Q^{A}\right)\log\left(I_{H_{A}}+Q^{A}\right),$$

which is equal to Equation (21).

The absolute convergence of the series Equation (21) follows from the estimate

(32) $$0\leq \left(1+\tau_{\alpha }^{2}\right)\log\left(1+\tau_{\alpha }^{2}\right)\leq 2\tau_{\alpha }^{2}.$$

The rest of the proof is obvious then. □

**Remark** **1.** *In finite dimensions, the von Neumann entropy is a non-negative, concave, lower semi-continuous, and also $L_{1}$—norm continuous function defined on the set of all quantum states. However, in the infinite dimensional setting, the conventionally defined von Neumann entropy takes the value +∞ on a dense subset of the space of quantum states of the system under consideration [26].*

*Nevertheless, the von Neumann entropy, defined in a standard way, has continuous and bounded restrictions to some special (selected by some physically motivated arguments) subsets of quantum states. For example, the set of states of the quantum oscillators system with bounded mean energy forms a set of states with finite entropy [26].*

*Very roughly, the reason for the infinite value of the von Neumann entropy effect in the infinite-dimensional setting is that in infinite dimensions there are many (too many!) sequences $\left(\lambda_{n}\right)$ such that: for all n, $\lambda_{n}\geq 0$ and $\sum_{n}\lambda_{n}=1$, but $\sum_{n}\log\left(\lambda_{n}\right)\lambda_{n}=-\infty$. In other words, the sets of states for which the values of the von Neumann entropy are (in)finite have no internal points and this fact causes serious problems in practice. The use of the Fredholm determinant technique* *[27,28], as described and proved in* *[25], leads to significant improvements in this field. In particular, the notion of the renormalised von Neumann entropy formula, as given in Equation (21), does arise in a natural way and leads to $L_{1}$–continuous functions Equation (31), which possess a lot of relevant properties, as expected for a good, infinite dimensional substitute for the standard von Neumann entropy.*

*The detailed, mathematical proofs of the results mentioned are under preparation for publication in a separate paper by one of the authors of the present paper [25].*

**Remark** **2.** *The kernels of the corresponding Equation (14) Schmidt expansions of the reduced density matrices $Q^{A}\left(\Psi \right)$ and $Q^{B}\left(\Psi \right)$ do not contribute to the corresponding entropies’ values. From this, it follows that the ambiguity, connected to the non-triviality of the kernels, is not essential in the process of reconstructing the state |Ψ〉 from a given $Q^{A}$ and $Q^{B}$ (see also the well known marginal problem [29]).*

## 3. Swapping Local Quantum Information (Slqi)

Let us consider two-partite system *A* and *B*, consisting of a *d*-dimensional qudits. The corresponding space of states of the system under consideration will be denoted as

(33) $$E\left(H^{A}⊗H^{B}\right)=\left\{Q:Q\geq 0\;\mathrm{and}\;\mathrm{Tr}\left(Q\right)=1\right\},\;H^{A}\approx H^{B}≃C^{d}.$$

With the qudits *A* and *B*, we associate the corresponding observers which, for a given global quantum state *Q*, have at their disposal only information contained on the corresponding reduced density matrices (termed RDMs) defined usually as

(34) $$Q^{A}={\mathrm{Tr}}_{B}\left(Q\right),$$

for observer $O^{A}$ and

(35) $$Q^{B}={\mathrm{Tr}}_{A}\left(Q\right),$$

for observer $O^{B}$.

**Definition** **1.** *An operation Sw:*

(36) $$\mathrm{Sw}:E\left(H^{A}⊗H^{B}\right)\to E\left(H^{B}⊗H^{A}\right),$$

*will be called a swapping local information operation for a given $Q\in E(H^{A}⊗H^{B})$ if the following equalities are true:*

(37) $${\mathrm{Tr}}_{B}\left(\mathrm{Sw}(Q)\right)=Q^{B}\;\mathrm{and}\;{\mathrm{Tr}}_{A}\left(\mathrm{Sw}(Q)\right)=Q^{A}.$$

**Figure 1:**
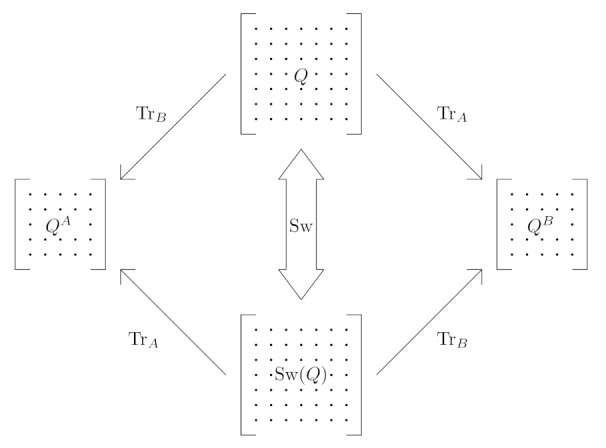
A graphical representation of the SWAP operation. Local information exchange is performed on the initial state *Q* (the final state, after the operation, is Sw(Q)). One can observe the swapping of subsystems *A* and *B* what may be calculated by the operation of partial trace.

**Remark** **3.** *Let us emphasise once more that swapping operations (as depicted in Figure 1) play a very important role in the constructions of the so-called quantum repeaters, which seems to be one of the important ingredients of long-distance quantum networks for quantum key distributions protocols realisation.*

The first question appearing is whether the set of SLQI for a given *Q* is non-empty. In order to mathematically construct such an operation (protocol), we proceed in the following way.

For a given $Q\in E(C^{d}⊗C^{d})$, let the corresponding spectral decomposition of *Q* be

(38) $$Q=\sum_{j=1}^{d^{2}}\lambda_{j}|\psi_{j}\rangle \langle \psi_{j}|,$$

where $\lambda_{j}\geq 0$, $\sum \lambda_{j}=1$ are the corresponding eigenvalues and the operators $E_{j}=|\psi_{j}\rangle \langle \psi_{j}|$ form the complete $\left(\sum E_{j}=I_{H^{A}⊗H^{B}}\right)$ and orthogonal system of projectors, that is, $\left(E_{i}⊥E_{j}=E_{i}E_{j}=\delta_{ij}E_{i}\right)$ onto the eigenvectors $|\psi_{j}\rangle$.

Now, for $j=1\;:\;d^{2}$, we can use the canonical Schmidt’s decompositions of the eigenstates $|\psi_{j}\rangle$:

(39) $$|\psi_{j}\rangle =\sum_{\alpha =1}^{d}\tau_{j}^{\alpha }|\varphi_{j}^{\alpha }\rangle ⊗|\theta_{j}^{\alpha }\rangle ,\;\tau_{j}^{\alpha }\geq 0,\;\sum_{\alpha =1}^{d}{\left(\tau_{j}^{\alpha }\right)}^{2}=1,$$

and the systems $|\varphi_{j}^{\alpha }\rangle \in H^{A}$, $|\theta_{j}^{\alpha }\rangle \in H^{B}$ of vectors form a complete orthonormal system of vectors in the corresponding Hilbert spaces.

Using Equation (39), we can obtain the formulas:

(40) $$Q^{A}=\sum_{j=1}^{d^{2}}\lambda_{j}Q_{j}^{A},\;\mathrm{where}\;Q_{j}^{A}=\sum_{\alpha =1}^{d}{\left(\tau_{j}^{\alpha }\right)}^{2}|\varphi_{j}^{\alpha }\rangle \langle \varphi_{j}^{\alpha }|,$$

and

(41) $$Q^{B}=\sum_{j=1}^{d^{2}}\lambda_{j}Q_{j}^{B},\;\mathrm{where}\;Q_{j}^{B}=\sum_{\alpha =1}^{d}{\left(\tau_{j}^{\alpha }\right)}^{2}|\theta_{j}^{\alpha }\rangle \langle \psi_{j}^{\theta }|.$$

Now, for each $j=1\;:\;d^{2}$, we define the unitary operators:

(42) $$\begin{array}{c} {\tilde{U}}_{j}^{AB}:H^{A}⟶H^{B},\;\;\;{\tilde{U}}_{j}^{AB}|\varphi_{j}^{\alpha }\rangle =|\theta_{j}^{\alpha }\rangle , \end{array}$$

for α=1:d, and extended by linearity to the whole space $H^{A}$. Similarly, we define the unitary operations:

(43) $$\begin{array}{c} {\tilde{U}}_{j}^{BA}:H^{B}⟶H^{A},\;\;\;{\tilde{U}}_{j}^{BA}|\theta_{j}^{\alpha }\rangle =|\varphi_{j}^{\alpha }\rangle , \end{array}$$

for α=1:d.

Then we define local unitaries:

(44) $$U_{j}^{AB}={\tilde{U}}_{j}^{AB}⊗I_{B},$$

and

(45) $$U_{j}^{BA}=I_{A}⊗{\tilde{U}}_{j}^{BA},$$

for α=1:d.

From the very definition, we have:

(46) $$\left(U_{j}^{AB}⊗U_{j}^{BA}\right)\left(|\varphi_{j}^{\alpha }\rangle \langle \varphi_{j}^{\beta }\left|⊗\right|\theta_{j}^{\alpha }\rangle \langle \theta_{j}^{\beta }|\right)\left({\left(U_{j}^{AB}\right)}^{†}⊗{\left(U_{j}^{BA}\right)}^{†}\right)=|\theta_{j}^{\alpha }\rangle \langle \theta_{j}^{\beta }\left|⊗\right|\varphi_{j}^{\alpha }\rangle \langle \varphi_{j}^{\beta }|.$$

For shorthand, we define:

(47) $$U_{j}=U_{j}^{AB}⊗U_{j}^{BA},$$

and let $E_{j}$ be the orthogonal projector onto the eigenvector $|\psi_{j}\rangle$. Then:

(48) $$\begin{array}{ccc} \mathrm{Sw}(Q) & = & \sum_{j=1}^{d^{2}}\lambda_{j}U_{j}E_{j}U_{j}^{†}\\ \; & = & \sum_{j=1}^{d^{2}}\lambda_{j}\sum_{\alpha =1,\beta =1}^{d}U_{j}\left(|\varphi_{j}^{\alpha }\rangle \langle \varphi_{j}^{\beta }\left|⊗\right|\theta_{j}^{\alpha }\rangle \langle \theta_{j}^{\beta }|\right)U_{j}^{†}\\ \; & = & \sum_{j=1}^{d^{2}}\lambda_{j}\left(\sum_{\alpha =1,\beta =1}^{d}\tau_{j}^{\alpha }\tau_{j}^{\beta }|\theta_{j}^{\alpha }\rangle \langle \theta_{j}^{\beta }\left|⊗\right|\varphi_{j}^{\alpha }\rangle \langle \varphi_{j}^{\beta }|\right). \end{array}$$

Let us observe that defining the vectors:

(49) $$|\psi_{j}^{\mathrm{Sw}}\rangle =\sum_{\alpha =1}^{d}\tau_{j}^{\alpha }|\theta_{j}^{\alpha }\rangle |\varphi_{j}^{\alpha }\rangle ,$$

for $j=1\;:\;d^{2}$, we obtain

(50) $$\begin{array}{cc} \langle \psi_{i}^{\mathrm{Sw}}|\psi_{j}^{\mathrm{Sw}}\rangle & =\sum_{\alpha ,\beta =1}^{d}\tau_{i}^{\alpha }\tau_{j}^{\beta }\langle \theta_{i}^{\alpha }|\theta_{j}^{\beta }\rangle \langle \varphi_{i}^{\alpha }|\varphi_{j}^{\beta }\rangle \\ \; & \\ \; & =\sum_{\alpha ,\beta =1}^{d}\tau_{i}^{\alpha }\tau_{j}^{\beta }\langle \varphi_{i}^{\alpha }|\varphi_{j}^{\beta }\rangle \langle \theta_{i}^{\alpha }|\theta_{j}^{\beta }\rangle \\ \; & \\ \; & =\langle \psi_{i}|\psi_{j}\rangle =\delta_{ij}, \end{array}$$

which means that the formula

(51) $$\mathrm{Sw}\left(Q\right)=\sum_{j=1}^{d^{2}}\lambda_{j}|\psi_{j}^{\mathrm{Sw}}\rangle \langle \psi_{j}^{\mathrm{Sw}}|,$$

gives the spectral decomposition of the operator Sw(Q) which is still non-negative and of trace equal to one.

Now, from Equation (51), it follows, easily (see Equations (40) and (41)) that

(52) $${\left(\mathrm{Sw}\left(Q\right)\right)}^{A}={\mathrm{Tr}}_{B}\left(\mathrm{Sw}(Q)\right)=\sum_{j=1}^{d^{2}}\lambda_{j}\left(\sum_{\alpha =1}^{d}{\left(\tau_{j}^{\alpha }\right)}^{2}|\theta_{j}^{\alpha }\rangle \langle \theta_{j}^{\alpha }|\right)=Q^{B}.$$

Similarly, we can prove that

(53) $${\left(\mathrm{Sw}\left(Q\right)\right)}^{B}={\mathrm{Tr}}_{A}\left(\mathrm{Sw}(Q)\right)=Q^{A}.$$

Thus, we have proved the following theorem:

**Theorem** **1.** *For any $Q\in E(C^{d}⊗C^{d})$ there exists a quantum operation Sw such that*

(54) $${\mathrm{Tr}}_{B}\left(\mathrm{Sw}(Q)\right)={\mathrm{Tr}}_{A}\left(Q\right)\;\;\;\mathrm{and}\;\;\;{\mathrm{Tr}}_{A}\left(\mathrm{Sw}(Q)\right)={\mathrm{Tr}}_{B}\left(Q\right)=Q^{A}.$$

**Proof.**  It follows from Equation (48). □

The corresponding to the operators $Q_{j}^{A(B)}$ (in the formulas Equations (40) and (41)) values of the von Neumann entropies are easy to calculate:

(55) $$\mathrm{for}\;j=1:d^{2}\;:\;\;\;S\left(Q_{j}^{A}\right)=S\left(Q_{j}^{B}\right)=-2\sum_{\alpha =1}^{d}{\left(\tau_{j}^{\alpha }\right)}^{2}\log\left(\tau_{j}^{\alpha }\right).$$

In the case of mixed states, the entropies of the corresponding reduced density matrices $Q^{A}$ and $Q^{B}$ are different in general:

(56) $$S\left(Q^{A}\right)=-\sum_{k=1}^{d}\Delta_{k}^{A}\log\left(\Delta_{k}^{A}\right)\neq -\sum_{k=1}^{d}\Delta_{k}^{B}\log\left(\Delta_{k}^{B}\right)=S\left(Q^{B}\right),$$

where $\Delta_{k}^{A(B)}$ are the corresponding eigenvalues of the reduced density matrix $Q^{A}$, resp. $Q^{B}$.

From the concavity of the von Neumann entropy, it results

(57) $$\sum_{j=1}^{d^{2}}\lambda_{j}S\left(Q_{j}^{A}\right)\leq -\sum_{k=1}^{d}\Delta_{k}^{A}\log\left(\Delta_{k}^{A}\right).$$

And similarly for part *B*:

(58) $$\sum_{j=1}^{d^{2}}\lambda_{j}S\left(Q_{j}^{B}\right)\leq -\sum_{k=1}^{d}\Delta_{k}^{B}\log\left(\Delta_{k}^{B}\right).$$

The following estimate

(59) $$\underset{\lambda ,\tau }{\sup}\left(-\sum_{j=1}^{d^{2}}\lambda_{j}\left(\sum_{\alpha =1}^{d}{\left(\tau_{j}^{\alpha }\right)}^{2}\log{\left(\tau_{j}^{\alpha }\right)}^{2}\right)\right)\leq \log\left(d\right),$$

is derived from the obvious estimate:

(60) $$\underset{Q\in E(C^{d}⊗C^{d})}{\sup}S\left(Q^{A}\right)\leq \log\left(d\right).$$

From the sub-additivity of the von Neumann entropy, we obtain:

(61) $$\left|S\left(Q^{A}\right)-S\left(Q^{B}\right)\right|\leq S\left(Q\right)\leq S\left(Q^{A}\right)+S\left(Q^{B}\right)\leq 2\log d.$$

**Proposition** **2.** *Let $Q\in E(C^{d}⊗C^{d})$ be a separable state. Then*

(62) $$-\sum_{j=1}^{d^{2}}\lambda_{j}\left(\sum_{\alpha =1}^{d}{\left(\tau_{j}^{\alpha }\right)}^{2}\log{\left(\tau_{j}^{\alpha }\right)}^{2})\right)\leq -\sum_{j=1}^{d^{2}}\lambda_{j}\log\lambda_{j}.$$

**Proof.**  Let sp(Q), $sp^{*}\left(Q^{A}\right)$ and $sp^{*}\left(Q^{B}\right)$ stand for (the ordered in the non-increasing order) lists of the corresponding eigenvalues. The $sp^{*}$ means that the corresponding lists are completed with an appropriate number of zeros (in fact $d^{2}-d$ zeros ). From the Nielsen-Kempe theorem [30], it follows that, for *Q* being separable, the majorization relations are valid:

(63) $$sp\left(Q\right)≺sp^{*}\left(Q^{A}\right)\;\;\;\mathrm{and}\;\;\;sp\left(Q\right)≺sp^{*}\left(Q^{B}\right),$$

where ≺ is the standard majorization relation, see for example, [8]. From the fact that the von Neumann entropy is a γ-monotone function, the following inequality emerges:

(64) $$\max\left(S\left(Q^{A}\right),S\left(Q^{B}\right)\right)\leq S\left(Q\right).$$

Using the decomposition Equations (40) and (41) together with concavity Equation (59), we obtain the above result. □

**Remark** **4.** *The obtained separability criterion in the case of pure states is exact. However, in the general case of mixed states, its domain of effective action is rather weak compared with many other separability versus non-separability criteria known in the literature. The advantage of the inequality Equation (62) is that it refers only to the basic, spectral data of a state under consideration.*

*Some applications of the obtained separability test are included in the next section of the paper.*

## 4. Quantum Switch as Unitary Local Information Swapping

The work [31] presents the idea of a quantum switch as a three-qubit controlled swap gate. Let us describe an initial state of this quantum system as:

(65) $$|\Psi_{qs}\rangle =|A\rangle |B\rangle |C\rangle ,$$

where the first qubit |A〉 and the second one |B〉 are unknown quantum qubit states, and we assume that the states |A〉 and |B〉 are presented as:

(66) $$|A\rangle =\alpha_{0}|0\rangle +\beta_{0}|1\rangle ,\;\;\;|B\rangle =\alpha_{1}|0\rangle +\beta_{1}|1\rangle ,$$

where $\alpha_{0},\beta_{0},\alpha_{1},\beta_{1}\in C$ and ${|\alpha_{0}|}^{2}+{|\beta_{0}|}^{2}=1$, ${|\alpha_{1}|}^{2}+{|\beta_{1}|}^{2}=1$.

The third qubit |C〉 is also called a controlling qubit and it accepts only one of two states |0〉 or |1〉. In general, the quantum switch can be regarded as a controlled SWAP gate. The mentioned gate swaps the states |A〉 and |B〉 according to a state of the qubit |C〉.

The way of operating for the quantum switch may be described by two cases. The first case takes place when the state of the qubit |C〉 is |0〉:

(67) |A〉|B〉|0〉⇒|A〉|B〉|0〉.

The quantum switch does not swap the states of input qubits. The operation of swapping is connected with the second case when the state of the qubit |C〉 is |1〉:

(68) |A〉|B〉|1〉⇒|B〉|A〉|1〉,

as it can be seen in Equation (68), the states of qubits |A〉 and |B〉 were swapped.

**Figure 2:**
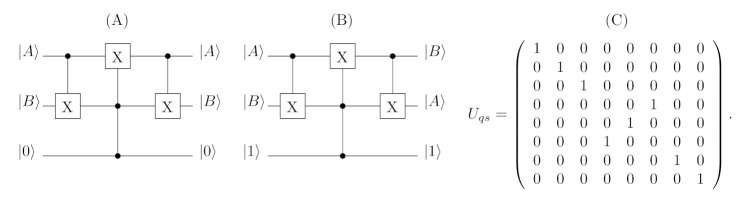
The circuits illustrating the operation of the quantum switch for qubits. If the state of the controlling qubit is |0〉 (case (A)) the switch does not change the order of first two input states. When the state of the third qubit is expressed as |1〉 (case (B)), the quantum switch swaps the input states |A〉 and |B〉. The matrix (C) represents the unitary operator of the switch operation.

A unitary operation corresponding to such behaviour needs to use only three quantum gates, that is, two controlled negation gates and the Toffoli gate (suitable definition of mentioned quantum gates can be found in [8]). Figure 2 depicts the quantum circuit realising operations performed by the quantum switch. Naturally, for a given matrix, forms of utilised gates, the unitary operation $U_{qs}$ (characterising the quantum switch) may be calculated directly. A definition of the unitary operator (in a permutation form) is as follows:

(69) $$U_{qs}=\left(\begin{array}{cccccccc} 1 & 0 & 0 & 0 & 0 & 0 & 0 & 0\\ 0 & 1 & 0 & 0 & 0 & 0 & 0 & 0\\ 0 & 0 & 1 & 0 & 0 & 0 & 0 & 0\\ 0 & 0 & 0 & 0 & 0 & 1 & 0 & 0\\ 0 & 0 & 0 & 0 & 1 & 0 & 0 & 0\\ 0 & 0 & 0 & 1 & 0 & 0 & 0 & 0\\ 0 & 0 & 0 & 0 & 0 & 0 & 1 & 0\\ 0 & 0 & 0 & 0 & 0 & 0 & 0 & 1 \end{array}\right).$$

Although the operation $U_{qs}$ captures the complete working of the switch, the evaluation of the entanglement level needs a Hamiltonian for a simpler presentation of system’s evolution. In this work, we define a simplified Hamiltonian’s form, because we can directly take advantage of the fact that the switch realises a swap operation only if the third qubit is in the state |1〉. That leads to the basic direct form of the Hamiltonian, describing the dynamics of the operation performed by the switch, where we reuse the Pauli X and Z operators applied in a subspace of the first and the second qubit with two additional couplings equal $\frac{1}{2}$:

(70) $$\begin{array}{c} H_{qs}=\frac{1}{2}\left(|011\rangle \langle 101|+|101\rangle \langle 011|\right)-\frac{1}{2}\left(|011\rangle \langle 011|+|101\rangle \langle 101|\right)=\\ \left(\begin{array}{cccccccc} 0 & 0 & 0 & 0 & 0 & 0 & 0 & 0\\ 0 & 0 & 0 & 0 & 0 & 0 & 0 & 0\\ 0 & 0 & 0 & 0 & 0 & 0 & 0 & 0\\ 0 & 0 & 0 & -\frac{1}{2} & 0 & \frac{1}{2} & 0 & 0\\ 0 & 0 & 0 & 0 & 0 & 0 & 0 & 0\\ 0 & 0 & 0 & \frac{1}{2} & 0 & -\frac{1}{2} & 0 & 0\\ 0 & 0 & 0 & 0 & 0 & 0 & 0 & 0\\ 0 & 0 & 0 & 0 & 0 & 0 & 0 & 0 \end{array}\right). \end{array}$$

The Hamiltonian $H_{qs}$, together with a time variable *t*, allows us to express the dynamics of the switch as a unitary time evolution operator:

(71) $$U_{qs}\left(t\right)=e^{-itH_{qs}},$$

and i represents the imaginary unit value.

A matrix form of the operator, for real values of *t* variable, is:

(72) $$U_{qs}\left(t\right)=\left(\begin{array}{cccccccc} 1 & 0 & 0 & 0 & 0 & 0 & 0 & 0\\ 0 & 1 & 0 & 0 & 0 & 0 & 0 & 0\\ 0 & 0 & 1 & 0 & 0 & 0 & 0 & 0\\ 0 & 0 & 0 & \frac{1+e^{\left(i\pi t\right)}}{2} & 0 & \frac{1-e^{\left(i\pi t\right)}}{2} & 0 & 0\\ 0 & 0 & 0 & 0 & 1 & 0 & 0 & 0\\ 0 & 0 & 0 & \frac{1-e^{\left(i\pi t\right)}}{2} & 0 & \frac{1+e^{\left(i\pi t\right)}}{2} & 0 & 0\\ 0 & 0 & 0 & 0 & 0 & 0 & 1 & 0\\ 0 & 0 & 0 & 0 & 0 & 0 & 0 & 1 \end{array}\right),$$

where t∈〈0,1〉, and for t=1 the switch correctly realises the swap operation for input states.

If the unitary operation Equation (72) is used, then the system’s state (with the control qubit in the state |0〉) may be expressed as:

(73) $$U_{qs}\left(t\right)|\Psi_{qs0}\rangle =\left(\begin{array}{c} \alpha_{0}\alpha_{1}\\ 0\\ \alpha_{0}\beta_{1}\\ 0\\ \alpha_{1}\beta_{0}\\ 0\\ \beta_{0}\beta_{1}\\ 0 \end{array}\right).$$

One can notice that there is no swapping of states. The gate $U_{qs}\left(t\right)$ does not perform any action on the quantum state. The action is performed when the state of the control qubit is |1〉:

(74) $$U_{qs}\left(t\right)|\Psi_{qs1}\rangle =|\Psi_{qs1}^{U_{qs}\left(t\right)}\rangle =\left(\begin{array}{c} 0\\ \alpha_{0}\alpha_{1}\\ 0\\ \frac{1}{2}\Lambda^{-}\left(t\right)\alpha_{1}\beta_{0}+\frac{1}{2}\Lambda^{+}\left(t\right)\alpha_{0}\beta_{1}\\ 0\\ \frac{1}{2}\Lambda^{+}\left(t\right)\alpha_{1}\beta_{0}+\frac{1}{2}\Lambda^{-}\left(t\right)\alpha_{0}\beta_{1}\\ 0\\ \beta_{0}\beta_{1} \end{array}\right),$$

where t∈〈0,1〉 and $\Lambda^{\pm }\left(t\right)=\left(1\pm e^{i\pi t}\right)$.

A density matrix, for the above pure state, is defined as:

(75) $$\rho_{qs1}\left(t\right)=|\Psi_{qs1}^{U_{qs}\left(t\right)}\rangle \langle \Psi_{qs1}^{U_{qs}\left(t\right)}|.$$

### 4.1. Quantum Circuit for $U_{Qs}\left(T\right)$ Operation

The Hamiltonian $H_{qs}$ (Equation 70) allows us to receive the unitary operator $U_{qs}\left(t\right)$. This operator has to be decomposed to the elementary gates set, to be implemented as a quantum circuit. Naturally, we can apply an approach based on the permutation operator Equation (69) of the switch but it would depict the whole process in one step. The $U_{qs}\left(t\right)$ decomposition to the set of elementary gates allows the construction of a circuit in which we can evaluate the entanglement level after each operation performed by a consequent gate. An exemplary circuit of quantum gates realising the switch is presented in Section 4.4.

Recently developed qiskit software [32] may be utilised for unsupervised decomposition of the operator $U_{qs}\left(t\right)$, preserving an influence of the parameter t∈〈0,1〉. For example, if $t=\frac{1}{4}$ then the circuit realises only quarter of the switch operating (in this case, the circuit’s realisation has to be repeated four times to perform the whole operation).

**Figure 3:**
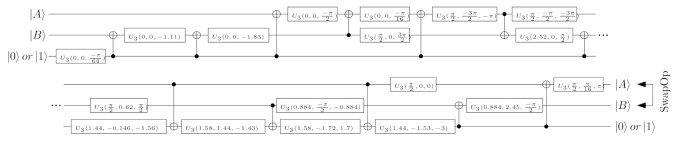
Decomposition of $U_{qs}\left(t\right)$ operation for t=1/4. The set of used gates enables the circuit’s implementation in qiskit and quantum machine IBM Q. Decompositions may be realised for arbitrary *t* what requires the changes in values of rotating gates U3 parameters.

Figure 3 shows an exemplary decomposition realised in qiskit. One can notice that it enables conversion of the switch to the set of basic gates automatically. In the proposed decomposition, only CNOT and U3 gates are utilised. The source code containing this decomposition is placed in the code repository dedicated for this article [33].

### 4.2. The Level of Entanglement for Switch

The main task of the switch is to transfer the information from the qubit *A* to the qubit *B*, which is enclosed in Equation (68). Tracing the process of information exchange between *A* and *B* is naturally connected through examining the entanglement level between these qubits. Of course, the entanglement may also be analysed for other pairs of qubits in the switch because introduced distortions may affect other parts of this system.

**Figure 4:**
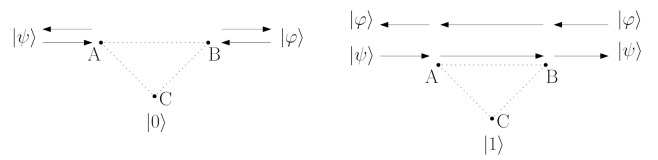
The examined entanglement level concerns mainly qubits *A* and *B*, which exchange their states (expressed as |ψ〉 and |φ〉) if controlling qubit C=|1〉. The entanglement level may also be analysed between pairs: A…C and B…C, which is marked above with the dotted line.

Figure 4 depicts the pairs of qubits in the switch. In this article, we examine the entanglement between qubits *A* and *B* when *C* is in state |1〉 for the case without and with a noise presence. To do this, we utilise the Negativity criterion for the two-qubit ρ state:

(76) $$N\left(\rho \right)=\frac{{\left|\right|\rho^{T_{A}}\left|\right|}_{1}-1}{2},$$

where $\rho^{T_{A}}$ represents a state after the partial transposition with respect to the first subsystem. The trace norm ${\left||X|\right|}_{1}$ of operator *X* can be expressed as:

(77) $${\left||X|\right|}_{1}=\mathrm{Tr}\left(|X|\right)=\mathrm{Tr}\left(\sqrt{X^{†}X}\right).$$

**Remark** **5.** *It should be added that a sum of singular values (obtained after the singular value decomposition) of X operator can be also used to compute a value of the trace norm.*

**Remark** **6.** *However, the most popular way to obtain the Negativity measure value is to calculate the absolute value of the sum of all negative eigenvalues $\lambda_{i}$ of the operator $\rho^{T_{A}}$:*

(78) $$N\left(\rho \right)=\sum_{\lambda_{i}<0}\left|\lambda_{i}\right|=\sum_{i}\frac{|\lambda_{i}|-\lambda_{i}}{2}.$$

*The above equation also shows that the negative eigenvalues may be gained as the difference between the absolute and relative value of the successive $\lambda_{i}$ (the range of values for i was omitted—the Negativity measure is used for a system of two qubits, so there are only four eigenvalues).*

According to Equation (76), it is necessary to calculate the following state:

(79) $$N\left(\rho_{qs}\left(t\right)\right)=N\left(\begin{array}{cccc} a{\left(a\right)}^{🟉} & a{\left(G\right)}^{🟉} & a{\left(H\right)}^{🟉} & a{\left(b\right)}^{🟉}\\ {\left(a\right)}^{🟉}\left(G\right) & \left(G\right){\left(G\right)}^{🟉} & \left(G\right){\left(H\right)}^{🟉} & {\left(b\right)}^{🟉}\left(G\right)\\ {\left(a\right)}^{🟉}\left(K\right) & \left(K\right){\left(G\right)}^{🟉} & \left(K\right){\left(H\right)}^{🟉} & {\left(b\right)}^{🟉}\left(K\right)\\ b{\left(a\right)}^{🟉} & b{\left(G\right)}^{🟉} & b{\left(H\right)}^{🟉} & b{\left(b\right)}^{🟉} \end{array}\right),$$

where $\rho_{qs}\left(t\right)$ symbolises a density matrix calculated as a partial trace operation, which erases |C〉=|1〉 from the system (the density matrix describes only qubit states A and B). We also use the substitutions:

(80) $$\begin{array}{c} a=\alpha_{0}\alpha_{1},b=\beta_{0}\beta_{1},c=\alpha_{0}\beta_{1},d=\alpha_{1}\beta_{0},\\ G=c\left(\frac{1}{2}+\frac{1}{2}e^{i\pi t}\right)+d\left(\frac{1}{2}-\frac{1}{2}e^{i\pi t}\right),\\ H=d\left(\frac{1}{2}+\frac{1}{2}e^{i\pi t}\right)+c\left(\frac{1}{2}-\frac{1}{2}e^{i\pi t}\right),\\ K=c\left(\frac{1}{2}-\frac{1}{2}e^{i\pi t}\right)+d\left(\frac{1}{2}+\frac{1}{2}e^{i\pi t}\right). \end{array}$$

The mentioned Negativity criterion allows us to formulate the following theorem:

**Theorem** **2.** *Let t be a real number and t∈〈0,1〉 (closed interval). The quantum switch, expressed as the unitary operation Equation (71) for the input state |AB1〉, introduces entanglement between qubits |A〉 and |B〉 for t∈(0,1) and there is no entanglement in moments t=0 and t=1.*

**Proof.**  The presence of an entanglement may be stated with the use of the Negativity measure. The vector state of the switch system is affected by the operator given in Equation (72) for particular *t*. Then, it is transformed to the density matrix (Equation (79)) where the partial trace operation to eliminate the qubit |C〉 (state of this qubit during the switch operating is |1〉) was performed. Next, the partial transposition according to the qubit |A〉 must be done, and the density matrix takes form:

(81) $$\begin{array}{c} \rho_{U_{sq}}^{T_{A}B}=\left(\begin{array}{cccc} a{\left(a\right)}^{🟉} & a{\left(G\right)}^{🟉} & {\left(a\right)}^{🟉}\left(K\right) & \left(K\right){\left(G\right)}^{🟉}\\ {\left(a\right)}^{🟉}\left(G\right) & \left(G\right){\left(G\right)}^{🟉} & b{\left(a\right)}^{🟉} & b{\left(G\right)}^{🟉}\\ a{\left(H\right)}^{🟉} & a{\left(b\right)}^{🟉} & \left(K\right){\left(H\right)}^{🟉} & {\left(b\right)}^{🟉}\left(K\right)\\ \left(G\right){\left(H\right)}^{🟉} & {\left(b\right)}^{🟉}\left(G\right) & b{\left(H\right)}^{🟉} & b{\left(b\right)}^{🟉} \end{array}\right), \end{array}$$

the marking $\rho^{T_{A}B}$ tells us that the system *A* is partially transposed, and the system *B* remains unchanged. We again use the following substitutions

(82) $$\begin{array}{c} a=\alpha_{0}\alpha_{1},b=\beta_{0}\beta_{1},c=\alpha_{0}\beta_{1},d=\alpha_{1}\beta_{0},\\ G=c\left(\frac{1}{2}+\frac{1}{2}e^{i\pi t}\right)+d\left(\frac{1}{2}-\frac{1}{2}e^{i\pi t}\right),\\ H=d\left(\frac{1}{2}+\frac{1}{2}e^{i\pi t}\right)+c\left(\frac{1}{2}-\frac{1}{2}e^{i\pi t}\right),\\ K=c\left(\frac{1}{2}-\frac{1}{2}e^{i\pi t}\right)+d\left(\frac{1}{2}+\frac{1}{2}e^{i\pi t}\right). \end{array}$$

Naturally, such a matrix has four eigenvalues, but only two of them (labelled as $n_{1}$ and $n_{2}$) may be negative numbers:

$$\begin{array}{c} \begin{array}{ccc} \lambda^{n_{1}} & = & \frac{1}{4}\sqrt{-1+e^{2i\pi t}}\left(\alpha_{0}\beta_{1}-\alpha_{1}\beta_{0}\right)\sqrt{\left(-1+e^{-2i\pi t}\right){\left(\alpha_{1}^{🟉}\beta_{0}^{🟉}-\alpha_{0}^{🟉}\beta_{1}^{🟉}\right)}^{2}},\\ \lambda^{n_{2}} & = & \frac{1}{4}\sqrt{-1+e^{2i\pi t}}\left(\alpha_{1}\beta_{0}-\alpha_{0}\beta_{1}\right)\sqrt{\left(-1+e^{-2i\pi t}\right){\left(\alpha_{1}^{🟉}\beta_{0}^{🟉}-\alpha_{0}^{🟉}\beta_{1}^{🟉}\right)}^{2}}. \end{array} \end{array}$$

After further algebraic transformations, the value of the Negativity measure may be expressed as:

(83) $$N\left(\rho_{U_{qs}}\left(t\right)\right)=\sqrt{\frac{{\left(\sin(t\pi )\right)}^{2}{\left|\alpha_{1}\beta_{0}-\alpha_{0}\beta_{1}\right|}^{4}}{4}}.$$

The above equation shows that the value of the Negativity measure is time-dependent. □

Equation (83) clearly shows that the values of amplitudes are constant. It is also easy to point out in which moments the entanglement vanishes, because of the basic properties of the sin function, for t=0 and t=1 the value of the Negativity measure equals zero.

**Corollary** **1.** *For the initial state |AA1〉, that is, states of the first two qubits are the same, Equation (83) illustrates that there is no entanglement in the system, because expression $a_{1}b_{0}-a_{0}b_{1}$ takes the form $a_{0}b_{0}-a_{0}b_{0}$, and equals zero.*

**Remark** **7.** *It should be emphasised that the level of entanglement is calculated for the system without distortions. The highest entanglement level, according to time t, is:*

(84) $$M_{Ent}\left(t\right)=\max_{t\in \langle 0,1\rangle }N\left(\rho_{U_{qs}}\left(t\right)\right)=\frac{1}{2}.$$

*After the analysis of the algebraic form of the Negativity value, we obtain that its maximum value appears in the moment $t=\frac{1}{2}$, which results from the basic properties of the sin function.*

**Figure 5:**
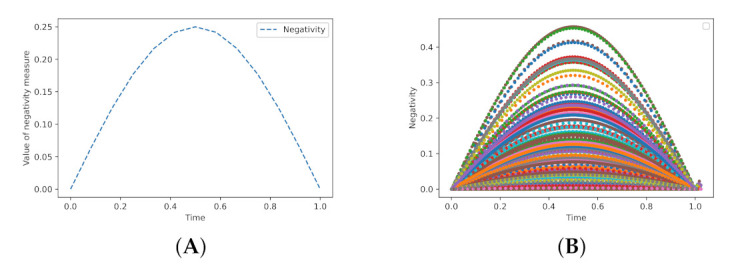
The chart (**A**) depicts the changes of the entanglement level between qubits A and B during the switch operating in time t∈〈0,1〉. States |A〉, |B〉 are described respectively as: |A〉=|+〉, |B〉=|0〉. Whereas, the chart (**B**) shows the values of the Negativity measure for the transfer of arbitrary selected states (128 states were used to create the chart (**B**)).

Figure 5 presents the changes in values of the Negativity measure during the switch operating, without any distortions. The first case concerns a specified state, and the second refers to arbitrary states produced by the pseudo random number generators.

**Figure 6:**
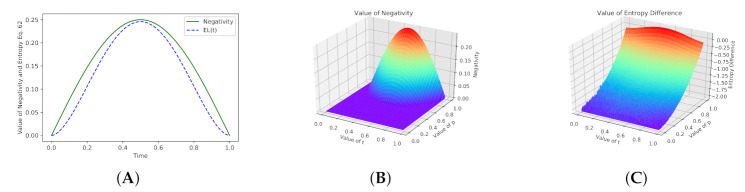
Values of the Negativity measure (**A**) and values calculated by the criterion Proposition (2), Equation (62) for the switch during its operating on states |A〉=|+〉, |B〉=|0〉. It is clear that both criteria evaluate the entanglement levels for pure states. The values of EL(t) are obtained as the differences between both sides of inequality Equation (62). It means that only for t=0 and t=1 both sides of the inequality are the same. In case (**B**), after the noise introduction (maximally mixed state), the Negativity measure still detects entanglement properly, but its level is decreased by the *p* value. Case (**C**) shows that EL(t) can be also used to indicate the presence of distortions by generating the negative values.

Equation (62) also allows us to evaluate an entanglement level in the switch for pure states, which is shown in Figure 6. The values from the chart (A) for EL(t) were calculated for $Q=\rho_{qs1}\left(t\right)$:

(85) $$EL\left(t\right)=\left(-\sum_{j=1}^{d^{2}}\lambda_{j}\left(\sum_{\alpha =1}^{d}{\left(\tau_{j}^{\alpha }\right)}^{2}\log{\left(\tau_{j}^{\alpha }\right)}^{2})\right)\right)-\left(-\sum_{j=1}^{d^{2}}\lambda_{j}\log\lambda_{j}\right).$$

The additional noise may be introduced as the maximally mixed state:

(86) $$\rho {\left(t\right)}_{qsmix}=\left(p\cdot \rho_{qs1}\left(t\right)\right)+\left(1-p\right)\frac{I}{8},$$

where p∈〈0,1〉 and I is the identity matrix sized 8×8. Unfortunately, when the extra noise is present, the criterion described in Equation (62) does not detect the entanglement properly. However, the left-hand side of the equation still indicates the changes in Entropy values because the switch is working (naturally, the value of Negativity measure points out the lack of entanglement for $p\leq \frac{1}{2}$). The chart (B) in Figure 6 shows changes in values of Negativity and Entropy EL(t) depending on parameter *p*.

### 4.3. The Level of Entanglement for Switch with Noise Presence

The switch during its operating is distorted by the Dzyaloshinskii–Moriya interaction (DMI) [34,35]. The applied interaction is described as:

(87) $$H_{DM}=\left(\sigma_{X}^{\left(i\right)}\sigma_{X}^{\left(i+1\right)}-\sigma_{Y}^{\left(i\right)}\sigma_{Y}^{\left(i+1\right)}\right).$$

The marking $\sigma_{X}^{\left(i\right)}$ tells us that the qubits, indexed as *i* and i+1, may be affected by one of the Pauli operators: X or Y. We introduce an additional real-valued parameter $D_{s}\in \langle 0,1\rangle$, which describes the strength of the interaction. The mentioned parameter may be utilised directly:

(88) $$H_{DM}\left(D_{s}\right)=D_{s}\cdot \left(\sigma_{X}^{\left(i\right)}\sigma_{X}^{\left(i+1\right)}-\sigma_{Y}^{\left(i\right)}\sigma_{Y}^{\left(i+1\right)}\right).$$

To examine the DMI influence on the switch, we need a new Hamiltonian $H_{\mathrm{TOT}}$, which represents the dynamics of these two joined systems:

(89) $$H_{\mathrm{TOT}}=H_{qs}+D_{s}\cdot H_{\mathrm{DM}},$$

where *t* stands for the time and $D_{s}$ for the DMI strength.

Thus, we can construct a unitary operator $U_{qs}^{\mathrm{DM}}$ which is equivalent of $H_{\mathrm{TOT}}$:

(90) $$U_{qs}^{\mathrm{DMI}}\left(t,D_{s}\right)=e^{-i(t\cdot H_{qs}+D_{s}\cdot H_{\mathrm{DM}})}.$$

It should be stressed that for $D_{s}=0$, we obtain the operator describing only the switch’s operating. Just as before, the time variable *t* accepts values from the interval 〈0,1〉.

However, the influence of DMI is modelled by the following relation, which describes the intrinsic decoherence effect, where the state in a moment *t* is given by the Milburn Equation [36]:

(91) $$\rho {\left(t\right)}_{DMI}=\sum_{m,n}\exp\left(-\frac{\gamma t\pi }{2}{\left(\mu_{m}-\mu_{n}\right)}^{2}-i\left(\mu_{m}-\mu_{n}\right)t\pi \right)\cdot \langle \Xi_{m}\left|\rho \left(0\right)\right|\Xi_{n}\rangle |\Xi_{m}\rangle \langle \Xi_{n}|,$$

where $\mu_{m}$, $\mu_{n}$ stand for eigenvalues and $\Xi_{m}$, $\Xi_{n}$ for eigenvectors of $H_{\mathrm{TOT}}$ Hamiltonian. The symbol γ refers to the intrinsic decoherence rate.

Eigenvalues of the Hamiltonian $H_{TOT}$ take the form:

(92) $$\mu_{0}=-1,\mu_{1}=0,\mu_{2}=0,\mu_{3}=0,\mu_{4}=-2\cdot D_{s},\mu_{5}=-2\cdot D_{s},\mu_{6}=2\cdot D_{s},\mu_{7}=2\cdot D_{s},$$

and its eigenvectors are:

(93) $$\begin{array}{ccc} \Xi_{0}=\frac{1}{\sqrt{2}}(-|3\rangle +|5\rangle ), & \Xi_{1}=\frac{1}{\sqrt{2}}(|3\rangle +|5\rangle ), & \Xi_{2}=|4\rangle ,\\ \Xi_{3}=|2\rangle , & \Xi_{4}=\frac{1}{\sqrt{2}}(-|1\rangle +|7\rangle ), & \Xi_{5}=\frac{1}{\sqrt{2}}(-|0\rangle +|6\rangle ),\\ \Xi_{6}=\frac{1}{\sqrt{2}}(|1\rangle +|7\rangle ), & \Xi_{7}=\frac{1}{\sqrt{2}}(|0\rangle +|6\rangle ). \end{array}$$

**Figure 7:**
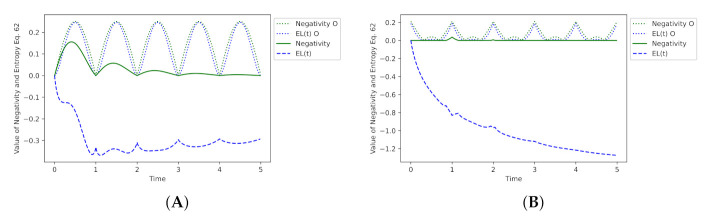
Plot (**A**) shows values of Negativity and EL(t) for a pure state, the dynamics of which are described by the operator $U_{qs}^{\mathrm{DMI}}$ without DMI distortions ($D_{s}=0$). Plot (**B**) presents entanglement levels when DMI is present and $D_{s}=0.25$ (again, the operator $U_{qs}^{\mathrm{DMI}}$ was used). On both graphs, the lines with the additional symbol O (Negativity O and EL(t) O) refer to cases without the intrinsic decoherence. Other lines depict the switch behaviour with the intrinsic decoherence for γ=0.5.

Figure 7 depicts the behaviour of the switch for some chosen values of γ and $D_{s}$. As we can see, distortions have a significant impact on the switch’s operating. Values of the entanglement are changed, and EL(t) values, based on the criterion Equation (62), point out that the density matrix is characteristic for a mixed state, if the intrinsic decoherence noise is added.

Values of the Negativity measure were calculated with respect to qubits A and B. EL(t) values were computed for the whole 3-qubit state of the switch. It should be emphasised that both Negativity and EL(t) can be applied to the tracking of entanglement level, if the quantum state of the system is pure.

If a state of the switch is mixed, then the Negativity measure correctly expresses a decrease in entanglement level, even in the presence of distortions. In the case of EL(t), its value does not point the entanglement but a mixed state, so we may say that the proposed criterion allows evaluation of whether the switch works properly, because for the operator $U_{qs}^{\mathrm{DMI}}$, the whole state of the register should be pure.

### 4.4. Quantum Circuit for Estimating the Level of Entanglement

**Figure 8:**
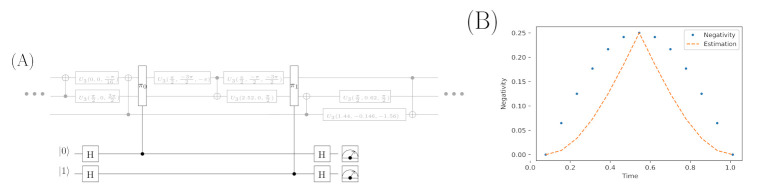
The scheme (**A**) of an exemplary quantum circuit estimating the entanglement level during switch’s operating in a chosen point of the quantum circuit. The gates $\pi_{0}$ and $\pi_{1}$ realise the controlled SWAP operation. The chart (**B**) shows the entanglement values estimated by the circuit (as the difference of probabilities calculated for measuring state |0〉 on both analysed qubits) and computed with the use of algebraic formula for the Negativity measure.

Figure 8 depicts the general circuit’s scheme, which may be utilised to the entanglement estimation between qubits A and B in the switch. Black elements of the circuit are responsible for the entanglement level estimation, while gray components symbolise elements which implement the switch. In general, the whole test is based on the approach of the SWAP-test [17] and properties discussed in [37]. The test is performed twice, that is, in moments when we want to estimate the entanglement’s values. A measurement of ancilla qubits is performed and the difference between the probabilities of measuring state |0〉 may be utilised for the Evaluation of Entanglement’s level (EE):

(94) $$EE\left(q_{4},q_{5}\right)=\left|p_{0}\left(q4\right)-p_{0}\left(q5\right)\right|,$$

where $q_{4}$, $q_{5}$ are the additional ancilla qubits and $p_{0}$ stands for the probability of measuring state |0〉.

It should be noted that the estimation is carried out without distortions and, naturally, it has a different scale than the Negativity measure but the mile stones of the entanglement level at the beginning, acme (t=1/2), and the end of the process, are properly imitated.

It is worth remembering that the entanglement estimating circuit allows us to observe its level only at one point. In addition, at least two extra qubits have to be used and, finally, the experiment has to be repeated to estimate the probability distribution properly. The values shown in Figure 8 are calculated by the black part of the circuit. However, the switch is implemented as the unitary operator decomposition $U_{qs}$ (obtained from the Hamiltonian $H_{qs}$), which is implemented as the circuit of elementary quantum gates and performs 1/12 of the whole switch operation. More details about the circuit’s implementation are captured in repositories of the source code for examples mentioned in Remark 8.

**Remark** **8.** *The values of entanglement, shown in this article, are calculated by the suitable Python scripts which may be downloaded from the Github repository [33].*

## 5. Conclusions

In this article, we have presented the quantum information transfer with the use of SWAP operation and its practical application as a circuit realising the quantum switch. A theoretical definition of the SWAP operation shows the exchange of quantum states in the system’s subspaces. Schmidt decomposition of quantum states allowed us to formulate the additional criterion, referring to Entropy, which may be utilized to evaluate the entanglement level during the switch’s operation.

We have described the realisation of the switch and analysed the level of entanglement during its work with the use of the Negativity measure and the proposed entropy-based criterion. The implementation of the switch is enabled owing to the presented Hamiltonian. The switch may be decomposed to a circuit of elementary quantum gates and we have shown how the SWAP-test may be utilised to evaluate the level of entanglement in a chosen point of the circuit.

Levels of entanglement in the switch were analysed in two cases: with and without distortions. Distortions are modelled in three ways: with the help of maximally mixed states matrix, as Dzyaloshinskii–Moriya interaction, and as the intrinsic decoherence effect described by the Milburn equation. One can see that the generated noise has an impact on the switch operating.

Values of the proposed criterion, for estimating the entanglement’s level, act similarly to values computed by the Negativity measure when there are no distortions caused by the intrinsic decoherence. If the intrinsic decoherence is present, we can still observe that a transition from EL(t) values to Negativity could be possible (e.g., multiplying EL(t) by (−1) causes curves to become more similar). It seems interesting to analyse transformations of EL(t), which could lead to Negativity values, and also to extract other needed transitions characteristic of other kinds of noise, which we postpone for future work in this area.

## Figures and Tables

**Table 1:** Symbols, states, and other denotations used in the paper.

Notation	Description
1..	sequence of integers from one to infinity
R	set of real numbers
C	set of complex numbers
N	set of integer numbers
U	set of unitary operators
H	complex Hilbert space
$H^{A}$, $H^{B}$	complex Hilbert space for *A* and *B* subsystems
I, $I_{d}$, $I_{H}$	unity operator, d-dimensional unity operator, and unity operator in Hilbert space H
*A*, *B*, *C*	single qubit or qudit
|A〉, |B〉, |C〉	pure state of single qubit or qudit
$|+\rangle =\frac{1}{\sqrt{2}}\left(|0\rangle +|1\rangle \right)$	positive superposition of base states |0〉 and |1〉
|3〉, |6〉, |7〉, *…*	pure state of quantum register, a state is described with decimal number
E(α,β)	projector on given state
*Q*	quantum state of the whole system
$Q^{A}$, $Q^{B}$	state of the subsystem *A* or *B*
$\delta_{\alpha \beta }$	Kronecker symbol
$\mathrm{Tr}\left(A\right)$	trace of matrix A
${\mathrm{Tr}}_{A}\left(Q\right)$	partial trace of system *Q*
Sw(·)	swap operation
S(·)	value of von Neumann Entropy
N(·)	value of Negativity criterion

## Data Availability

Not applicable.
